# Beyond Multiple Choice: The Association between Diagnostic Reasoning Exams and Medical Student Learning Strategies

**DOI:** 10.1007/s40670-026-02864-8

**Published:** 2026-08-28

**Authors:** Hope Cherian, Rachel Dockter, Rebecca L. Toonkel, Gagani Athauda, Jenny Fortun

**Affiliations:** 1 Florida International University Herbert Wertheim College of Medicine, 11200 SW 8th St AHC2, Miami, FL 33199 USA; 2 Department of Medical Education, Miami , USA

**Keywords:** Medical education, Multiple-choice exams, Diagnostic reasoning exams, Study strategies, Study resources

## Abstract

**Introduction:**

Assessment format shapes how medical students study. While multiple-choice questions (MCQ) are widely used, they may incentivize pattern recognition and memorization. We developed and implemented a novel open-ended assessment format, the Diagnostic Reasoning (DxR) exam, designed to mirror the step-by-step, iterative nature of clinical decision-making. Our goal was to verify content delivery validity and to study the effects of employing a hybrid assessment approach (MCQ and DxR) across multiple courses on students’ study strategies.

**Method:**

End-of-course DxR and MCQ exams were administered concurrently at the end of four second-year organ systems courses for the Classes of 2021–2024. Four independent expert raters reviewed each exam to generate item and scale level content validity indices. Students were surveyed about study resources and strategies used, and frequency of re-organizing study around presenting complaints and differential diagnoses rather than disease states for each assessment format.

**Results:**

Content validity analysis revealed strong alignment between content and intended construct for the DxR exam (S-CVI = 0.93). DxR exam preparation favored institution-developed resources, while MCQ preparation emphasized board-oriented materials, particularly question banks and review texts (*P* < .001, Cramér’s V = 0.26). Study strategies also differed significantly (*P* < .001, Cramér’s V = 0.19) with MCQ preparation favoring individual study, while DxR preparation included self-created cases, content reorganization, and group study. Learners were 2.4 times more likely to report organizing study material by clinical presentation rather than disease state when studying for DxR exams (*P* < .001).

**Conclusions:**

DxR exams demonstrate high content validity. Students report more frequent use of study strategies more closely aligned with institutional learning objectives and the development of clinical reasoning skills when studying for DxR exams than when studying for MCQ exams. Incorporating complementary assessment formats may align curricular objectives with both licensing preparation and diagnostic reasoning skill development.

**Supplementary Information:**

The online version contains supplementary material available at https://doi.org/10.1007/s40670-026-02864-8.

## Introduction

Medical education best practices emphasize the integration of clinical reasoning and foundational knowledge in both course content and assessment, as well as alignment between the two [1, 2]. According to constructive alignment theory, curriculum design, teaching methods, and assessment formats must be intentionally structured to support desired learning outcomes [1, 3]. Assessment thus functions not only as a measure of knowledge, but also has the potential to shape what, how and when students learn, particularly when coupled with learner support and meaningful feedback [2, 4]. When students perceive misalignment between course objectives and assessment formats, they may strategically adapt their study approaches to optimize performance on the assessment, rather than to master the intended competencies [5–7].

This dynamic between assessment and learning has been well-documented in medical education research. Qualitative and quantitative studies reveal that students select study methods based on their perception of what the assessment rewards, often prioritizing memorization and pattern recognition for multiple-choice question (MCQ) exams at the expense of deep learning and clinical integration [5, 6, 8, 9]. Students may even omit clinically relevant material if they perceive it as unlikely to appear on exams, highlighting how assessment format shapes not only knowledge acquisition, but also professional development [7, 8]. These findings underscore the essential role of assessment design in promoting higher-order reasoning and durable learning. Active learning approaches have been associated with improved long-term knowledge retention and exam performance [10, 11], further reinforcing the need for thoughtful alignment of assessment methods that encourage deep learning [4, 12].

MCQ exams remain the most frequently employed assessment format in pre-clerkship medical education because of their high reliability, efficiency, and broad content coverage [2, 3]. While some well-designed MCQs can assess higher-order clinical reasoning, they provide limited insight into students’ underlying thought processes [6, 13]. Constructed-response formats such as open-ended questions (OEQs) and case-based exercises, in contrast, require students to generate explanations, integrate concepts, and demonstrate clinical reasoning [14–19]. These formats have been shown to promote deeper learning [18, 19] and predict performance on clinical assessments [20–22] but may introduce reliability concerns and grading burden [23].

Given the complementary strengths of MCQs and OEQs, our institution employs a blended assessment model for pre-clerkship end-of-course examinations which includes a novel open-ended assessment format known locally as the “Diagnostic Reasoning Exam” (DxR). The DxR exam consists of a single progressive disclosure patient case requiring students to develop and refine a differential diagnosis, interpret findings, and justify management decisions [24]. An initial study of the DxR exam format in a single course demonstrated that 38% of students reorganized their study processes by clinical presentation rather than disease process, reflecting a meaningful shift in cognitive framing and study behavior [24]. This study extends that work by describing the refined DxR format, confirming content validity, and implementing the strategy across multiple courses and cohorts to conduct more in-depth analyses. Here we further explore specific questions about student study behaviors and resources used for exam preparation, and assess the generalizability and broader curricular relevance of these findings across multiple courses and student cohorts. We hypothesized that students would rely more heavily on locally developed resources and favor study strategies more aligned with the development of clinical reasoning skills when preparing for DxR exams than when preparing for MCQ exams.

## Methods

### Participants

Second-year students in the Classes of 2021 through 2024 who completed end-of-course surveys from the Cardiovascular & Pulmonary, Gastrointestinal, Reproductive, and Nervous System courses at our institution were included in this study (118 students in 2021, 119 students in 2022, 128 students in 2023, and 110 students in 2024). Survey response rate ranged from 68 to 91% by course. This study was approved by the local Institutional Review Board (#22–0250).

### Assessments

We evaluated 28 end-of-course MCQ and DxR assessments (14 paired) administered in the Cardiovascular & Pulmonary, Gastrointestinal, Reproductive, and Nervous System courses from 2021 to 2024. No data was available for the Cardiovascular & Pulmonary course in 2022 or the Nervous System course in 2021.Each course was assessed with one MCQ exam, worth 40% of the final grade, and one DxR exam, worth 10% of the final grade, administered to the same cohort simultaneously or within 24 h. Together, these exams accounted for 50% of the final course grade, with the remaining portion derived from other course activities earlier in the term.

*MCQ examinations.* MCQ exams are constructed by course directors and course faculty using the National Board of Medical Examiners (NBME) Customized Assessment Service (CAS). Each MCQ exam includes approximately 100 questions with a mix of application and recognition-style questions, and accounts for approximately 40% of the final course grade. MCQ exams were similar across all cohorts for each course, with 80–100% of exam questions remaining unchanged during the study period.

*Diagnostic reasoning examinations.* DxR exams and associated grading rubrics are constructed by course directors and content-expert faculty and are reviewed by a copy editor for grammar and by at least three members of the institution’s standing Exam Review Committee for consistency and accuracy. Grading is performed by trained faculty familiar with the exam style, with each question scored by a single grader to prevent variability that could be introduced by multiple raters. Each DxR exam presents a single patient case with 6 to 14 OEQs and accounts for 10% of the final course grade. Students advance through the DxR exam sequentially to simulate a patient encounter, receiving new information, and answering additional OEQs at each step. Backwards navigation is not permitted, but all prior and new information is available for review. After several iterations since its first description [24] the exam format was progressively refined based on student and faculty feedback, resulting in the development of a new standardized format that has continued to be used over time (Supplementary Material 1):

- Part 1 requires students to develop a list of 5 plausible diagnosis and for each one, include follow-up questions and predicted physical exam findings.
- Part 2 requires students to narrow and justify their differential (top 3) based on additional information.
- Part 3 requires further refinement of the differential and proposal of diagnostic tests and expected results based on physical exam findings.
- Part 4 provides diagnostic results for interpretation and selection of the final diagnosis with supporting elements from all available information.
- Subsequent sections may vary, but in all cases final questions assess understanding of disease pathophysiology and therapeutic decision-making, including correlation with foundational knowledge.

To minimize potential familiarity with examination content, the same exam was not administered in consecutive years. Reused examinations were typically separated by approximately three years. Although the format and type of questions of the DxR were discussed with the class, potential cases were not announced. *Formative feedback*: Students received an orientation to DxR exams during the first course of the organ system block where one entire case and grading rubric were discussed with the class, as well as the format and expectations for these exams. Additionally, students had access to practice exams and practice questions. At the end of each DxR exam, students were able to review their answers together with a model answer developed by the faculty.

### Outcomes

#### Exam Performance

 Exam performance was calculated based on average percent correct.

#### DxR Exam Content Validity

To support interpretation of study strategy findings, we evaluated whether the DxR exam assessed diagnostic reasoning constructs using a structured expert review process. Four faculty members independently rated each exam on its representation of diagnostic reasoning according to published criteria [21] including information gathering, hypothesis generation, problem representation, differential diagnosis creation, identification of the leading diagnosis, diagnostic justification, management and treatment, and pathophysiology. Pathophysiology was added specifically for this study. Raters had access to the entire exam, feedback, and student responses. Ratings were recorded dichotomously, as present or not present.

#### Student Study Methods

Anonymous end-of-course surveys were administered through E-Value (MedHub, Minneapolis, MN) [25]. Informed by the prior study, the survey instrument included additional items and more in-depth questions about study resources and strategies used for DxR and MCQ exam preparation (Supplementary Material 2) [24]. For resources, students were asked to identify resource types most used when studying for each exam type (unranked, top four: Step 1 review books, Step 2 review books, lecture materials, Step 1 MCQ resources, Step 2 MCQ resources, other MCQ resources, cases and/or small-group reading material, reading material required for lecture and/or lab sessions, materials required for Clinical Skills and/or other institution-offered courses, and other resources). Step 2 resources were included as answer choices based on student feedback that these resources offer tailored diagnosis-focused information that allows for information consolidation in a unique way. For study strategies, students were asked to identify those used when studying for each exam type (all that apply: individual study, group study, content reorganization, self-created cases, and others). Finally, students were asked about the frequency of organizing study material by clinical presentation rather than disease state for each exam type (5-point Likert, 1-not at all through 5-a tremendous amount).

### Statistical Analyses

#### Exam Performance

Mean performance on MCQ and DxR exams was compared using paired t-tests. The association between MCQ and DxR grades was assessed using Pearson correlation.

#### DxR Exam Content Validity

To evaluate whether each question was representative of the construct, 4 expert faculty raters not involved with this analysis independently rated each exam item using previously described criteria for diagnostic reasoning [21]. It has been previously determined that four raters was adequate, based on studies which used two to twenty interdisciplinary experts [26]. Item-level content validity indices (I-CVI) were calculated as the proportion of raters indicating that each exam item assessed its intended diagnostic reasoning domain [27]. To evaluate whether the entire exam represents the construct, we calculated scale-level content validity indices (S-CVI) using two different scales – the S-CVI average (S-CVI/Ave), which is the overall agreement (average I-CVI) across all items, and the S-CVI universal agreement (S-CVI/UA), which is the proportion of items where all raters agreed that the item intended was measured by the question. Interrater reliability was assessed using Fleiss’ κ, to account for multiple raters providing categorical judgments [28].

#### Student Study Methods

Differences in reported resources and strategies between DxR and MCQ preparation were assessed using Chi-square tests, with Cramér’s V used to estimate effect size. Post-hoc analysis of standardized residuals were performed to identify categories contributing most to observed differences (i.e. over- or under-represented relative to expected frequencies). For study organization, Likert responses were dichotomized (1–3 vs. 4–5) to represent lower versus higher frequency of organizing material by clinical presentation. Paired responses were compared using the McNemar test.

All analyses were conducted using Microsoft Excel v2506 (Microsoft Corp, Redmond, Washington) [29] and Python 3.13 (Python Software Foundation, Wilmington, Delaware) [30]. Significance was set at *P* < .05.

## Results

### Exam Performance

Across cohorts, exam scores ranged widely from 70 to 90% correct without a consistent pattern indicating either exam format was more difficult than the other. Average student performance across all cohorts on both the DxR and MCQ exams was comparable (84.0%). Pearson correlation between DxR and MCQ ranged from 0.13 to 0.48, with an average of 0.34 for all exams, demonstrating low to moderate association between exam formats (Supplementary Material 3).

### DxR Exam Content Validity

**Fig. 1:**
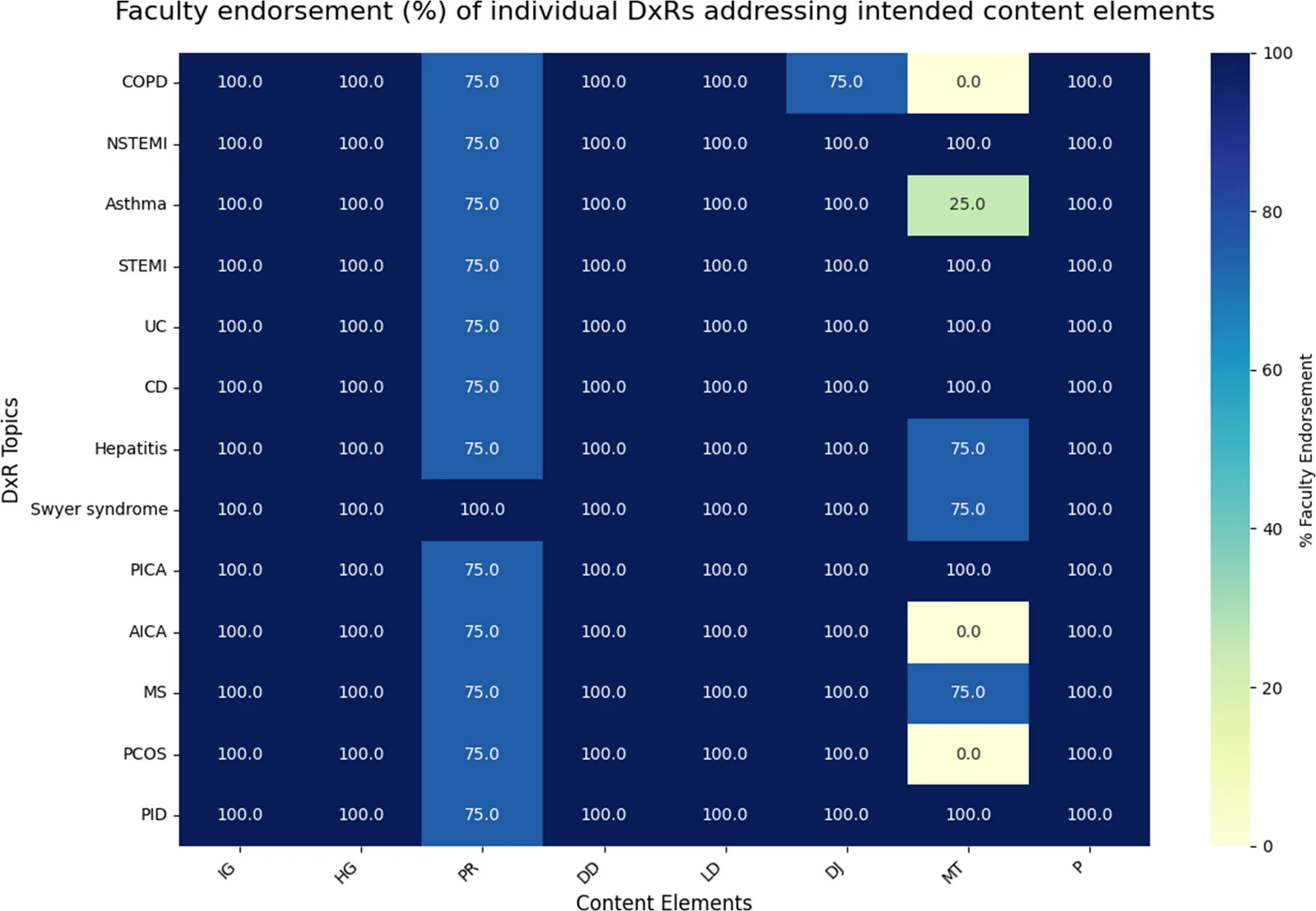
Content validity of Diagnostic reasoning exams: I-CVI heatmap indicating the percent of faculty reviewers agreeing that each exam item addressed intended content elements. Darker shading indicates higher agreement, with numeric values representing percent agreement (0-100%)

I-CVIs for each diagnostic reasoning domain on each DxR exam are shown in Fig. 1. All raters agreed on the inclusion and adequate representation (100% agreement) for “information gathering,” “hypothesis generation,” “differential diagnosis,” and “diagnostic justification,” and “pathophysiology,” while rater agreement was lower for “problem representation” and “management and treatment.” S-CVIs indicated acceptable overall validity of the entire exam (S-CVI/Ave = 0.92; S-CVI/UA = 0.81). Overall interrater agreement, as measured by Fleiss’ κ, was 0.41, reflecting moderate agreement among reviewers [27, 28].

Table 1 summarizes differences in study resources, strategies, and organizational approaches used for DxR versus MCQ exam preparation. Overall, students reported using different resources (χ² (8) = 647.1, *P* < .001; Cramér’s V = 0.26) and study strategies (χ² (4) = 169.8, *P* < .001; Cramér’s V = 0.19) based on the exam type. Additionally, students were more likely to organize their study by clinical presentation than disease state when preparing for DxR exams compared with MCQ exams (736 vs. 305, [χ² (1) = 55.96, *P* < .001]), representing a 2.4-fold difference.

**Table 1:** Patterns of study resources, strategies, and organization based on examination type

	Variables	DxR	MCQ
Study Resource,N (% within resource) ^a^	Lecture Materials	1020 (54.2)	861 (45.8)
	Course Readings	376 (58.2)	270 (41.8)
	Active Learning SessionMaterials	942 (66.9)	466 (33.1)
	Clinical Skills Course Materials	556 (77.7)	160 (22.3)
	Step 1 Review Books	971 (44.0)	1235 (56.0)
	Step 1 MCQ Resources	618 (34.5)	1173 (65.5)
	Step 2 Review Books	87 (73.7)	31 (26.3)
	Step 2 MCQ Resources	13 (39.4)	20 (60.6)
	Other	252 (42.3)	338 (57.3)
Study Strategy,N (% within strategy) ^b^	Group Study	656 (61.9)	404 (38.1)
	Content Reorganization	549 (65.5)	289 (34.5)
	Self-created Cases	187 (83.9)	36 (16.1)
	Individual Study	1228 (48.8)	1289 (51.2)
	Other	24 (50.0)	24 (50.0)
Study Organization Strategy,N (% within strategy) ^c^	Patient Presentation ^d^	736 (70.7)	305 (29.3)
	Disease State ^e^	521 (35.1)	962 (64.9)

^a^ χ²(8) = 647.1, *P* < .001; moderate effect size (Cramér’s V = 0.26)

^b^ χ²(4) = 169.8, *P* < .001; weak-to-moderate effect size (Cramér’s V = 0.19)

^c^ χ²(1) = 55.96, *P* < .001

^d^ Includes responses corresponding to Likert scale values 1-3 (“Not at all,” “A little bit,” “Somewhat”) for the question: “When organizing your study for the MCQ examination, how frequently did you start with patient presentations (signs and symptoms) and then learn the associated diseases?”

^e^ Includes responses corresponding to Likert scale values 4-5 (“Quite a bit,” “Very much”) for the same question.Notes: Percentages are calculated within each study resource/strategy because respondents could select multiple options; therefore, percentages across rows may sum to >100%

*Abbreviations*: *DxR* Diagnostic Reasoning, *MCQ* Multiple-choice Question

**Fig. 2:**
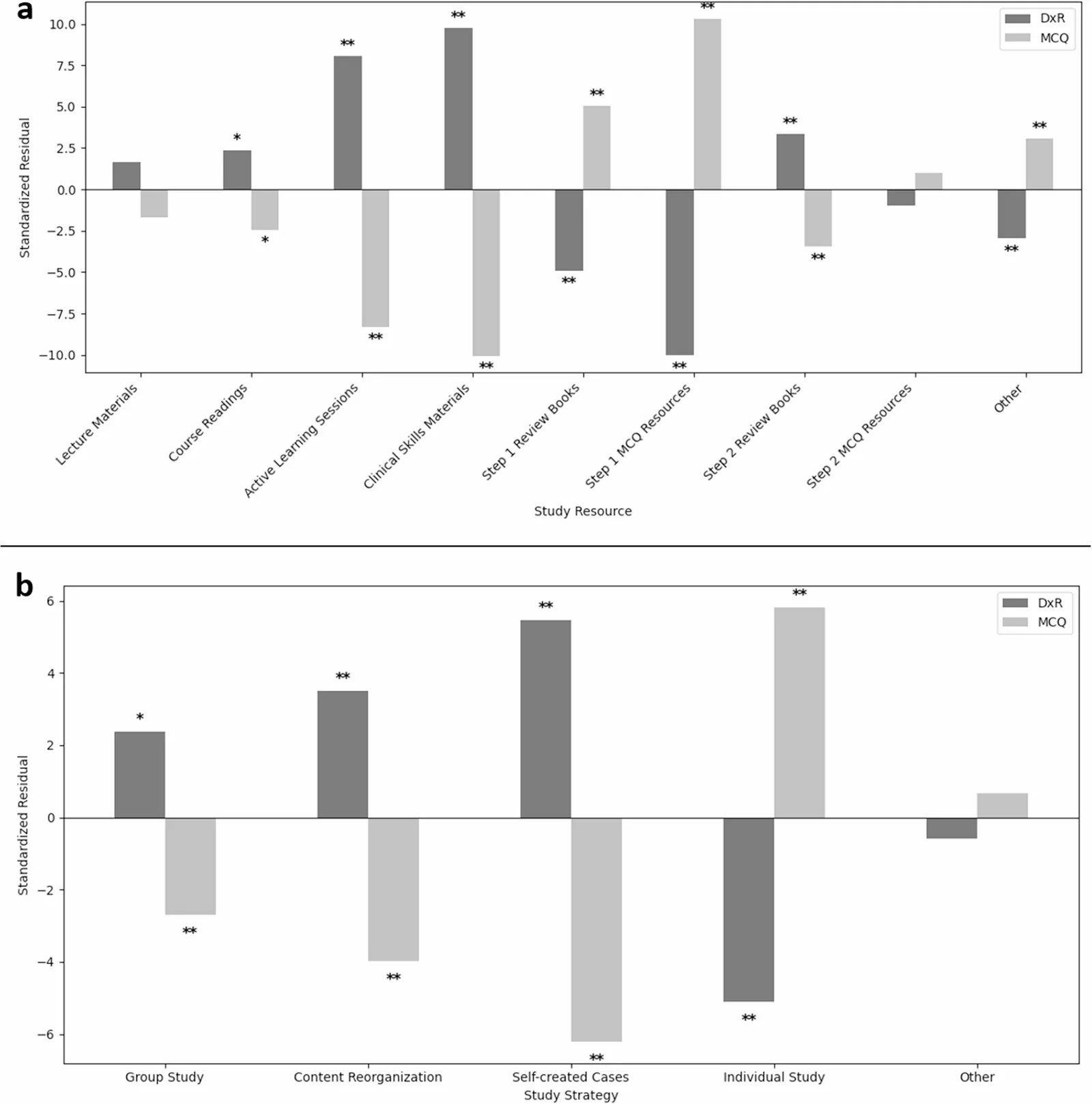
Utilization of study resources and strategies by exam type: Bars represent standardized residuals showing the difference in utilization of study resources (**a**) and study strategies (**b**) by exam type (DxR, dark grey vs. MCQ, light grey). Positive z-values (z > 0) indicate over-represented items, while negative z-values (z < 0) indicate under-representation. Significance thresholds: *|*z*| ≥ 1.96 (*P* < .05); **|*z*| ≥ 2.58 (*P* < .01)

Standardized residuals identified specific patterns driving differences in resource use (Fig. 2a) and study strategies (Fig. 2b). When studying for MCQ exams, students preferentially use outside resources including Step 1 MCQ (z = 10.3, *P* < .01) and Step 1 review books (z = 5.0, *P* < .01). “Other” resources were also used more for MCQ preparation, which according to students’ comments, largely represented Anki, an open-source flashcard program, and specific online resources and platforms aimed to Step 1 preparation. In contrast, studying for the DxR was associated with greater reliance on institutionally developed material that aligned with the school curriculum, including active learning sessions (z = 8.1, *P* < .01), course readings (z = 2.4, *P* < .05), and materials from the concurrent Clinical Skills course (z = 9.8, *P* < .01). As shown in Fig. 2b, individual study was more commonly employed to study for MCQ exams (z = 5.8, *P* < .01), whereas self-created cases (z = -6.21, *P* < .01) and content reorganization (z = -3.99, *P* < .01) were used less frequently. Conversely, DxR preparation was associated with greater use of group study (z = 2.37, *P* < .05), self-created cases (z = 5.45, *P* < .01), and content reorganization (z = 3.50, *P* < .01).

## Discussion

Assessment structure exerts a powerful influence on patterns of exam preparation [2, 12]. Because diagnostic reasoning is a learned skill that benefits from structured practice, including strategies such as self-explanation, case-based rehearsal, and reflection on reasoning errors, it stands to reason that assessment formats aligned with these strategies may encourage parallel study strategies [15–17]. 

Herein, we describe a novel OEQ assessment format (the DxR exam) designed to mirror the step-by-step and iterative nature of clinical decision-making, with the intention of better aligning students’ study approaches and consolidation of knowledge clinical skills development. We found a positive but modest correlation in student performance between the MCQ and DxR assessment formats, indicating that while the two exam formats test similar underlying knowledge, they also measure distinct skills, which may have complementary value in student assessment. As shown by content validity assessments, the DxR exam format exhibits strong alignment between item content and intended constructs for clinical reasoning [31], supporting its use in the assessment of diagnostic reasoning skills. Notably, not all exams included questions related to management, as some management decisions (e.g., surgical interventions), were not considered an expected competency for second-year medical students. Additionally, the discrepancy observed in this domain was attributed to the rating provided by the pharmacology expert, who may have had different expectations regarding the management knowledge assessed and/or the level of knowledge expected of pre-clerkship students. 

A growing body of evidence documents medical students’ increasing reliance on third-party board-preparation tools [7, 9]. While this “parallel curriculum” may help students to optimize performance on MCQ-based standardized exams, it does so at the expense of locally defined objectives. Consistent patterns emerged characterized by reliance on third-party board-preparation resources for MCQ exam preparation. On the other hand, students relied more heavily on institution-provided, case-based learning materials in preparation for DxR exams. Additionally, more students reported using resources from the concurrent, but distinct, Clinical Skills course to prepare for DxR exams, suggesting a move towards knowledge integration across courses. DxR studying also motivated students to create their own cases, shifting their role to active designers, which promotes a deeper understanding and requires further integration across different content areas. This is relevant, as integrated understanding of clinical and basic science knowledge within both curriculum and assessment supports the development of clinical reasoning and expertise [32]. The observed study pattern may be advantageous for re-engaging students with the institutional curriculum, promoting a more balanced approach that fosters comprehensive learning, knowledge integration, and the development of clinical reasoning skills.

Consistent with prior findings [24], we observed that organizing study content by patient presentation rather than by disease state for DxR exams persists across multiple courses and cohorts, suggesting that this pattern is reproducible and sustained longitudinally. The observed change in study strategies towards those that more closely mimic clinical practice supports the use of DxR-style exams to help students refine diagnostic reasoning skills, bridging the gap between pre-clerkship learning and clinical decision-making. Although the long-term impact of these habits was not measured, prior work suggests that practice with diagnostic case analysis facilitates application of knowledge to novel clinical problems and may support performance in subsequent clinical settings [20]. 

### Limitations

While the results of this study suggest changes in study behavior (Kirkpatrick Level III) [33] based on exam type, they do not establish causal changes in students’ study habits or guarantee longer-term impacts such as improved clinical performance or diagnostic reasoning skills. Moreover, the percentage agreement for a given strategy may have been influenced by the different contributions of MCQ and DxR exams to the final course grade (40 vs. 10 percent). Because the two exams occurred within 24 hours of one another, the study design did not include an analysis of each assessment independently, limiting our ability to determine whether DxR exams independently promote greater integration of concepts or application of knowledge. We purposely did not assess whether the pattern of study influenced performance because of the limitation of both exams having been administered together, thus necessarily causing the study patterns for one exam to influence performance on the other. Accordingly, our results should not be interpreted as a comparison of MCQ with DxR exams. Rather, we describe how students adapt their study habits to meet the varied learning requirements in a hybrid assessment model, and how this pattern can foster engagement with curriculum content. Although the long-term impact of these habits was not measured, prior work suggests that practice with diagnostic case analysis facilitates application of knowledge to novel clinical problems and may support performance in subsequent clinical settings [20]. Further research would benefit from examining whether these study approaches translate to performance in clinical settings.

## Conclusion

This study confirms content validity of the DxR exam format and demonstrates that students preparing for DxR exams report increased frequency of use of curriculum-based learning resources rather than reliance on third-party material. Furthermore, the increased frequency of organizing study content by patient presentation rather than by disease state for DxR exams persists longitudinally across multiple courses and multiple cohorts, suggesting that this pattern is reproducible and sustained longitudinally. The observed shift in study strategies towards approaches that more closely resemble clinical practice, supports the potential use of DxR-style exams to help students refine diagnostic reasoning skills, bridging the gap between pre-clerkship learning and clinical decision-making. Overall, the introduction of the DxR exam may promote content integration and generative reasoning, and importantly, re-engage students with the school’s curriculum. 

## Supplementary Information

Below is the link to the electronic supplementary material.

Supplementary Material 1.

## Data Availability

Data is available by request to the Corresponding Author.

## Abbreviations

DxR: Diagnostic Reasoning
IG: Information gathering
HG: Hypothesis generation
PR: Problem representation
DD: Differential diagnosis
LD: Leading diagnosis
DJ: Diagnostic justification
MT: Management and treatment
P: Pathophysiology
COPD: Chronic obstructive pulmonary disease
NSTEMI: Non-ST elevation myocardial Infarction
STEMI: ST-elevation myocardial infarction
UC: Ulcerative Colitis
CD: Crohn’s Disease
PICA: Posterior Inferior Cerebellar Artery stroke syndrome
AICA: Anterior Inferior Cerebellar Artery stroke syndrome
MS: Multiple Sclerosis
PCOS: Polycystic Ovarian Syndrome
PID: Pelvic Inflammatory Disease

## Declarations

### Disclaimers

None declared.
