# Supplementary material for: Beyond Multiple Choice: The Association between Diagnostic Reasoning Exams and Medical Student Learning Strategies
Source: Med Sci Educ. 2026 Aug 28;36(6):2983–91. doi: 10.1007/s40670-026-02864-8 (PMC13759882; doi:10.1007/s40670-026-02864-8)
Supplement: Supplementary file 1 [file 40670_2026_2864_MOESM1_ESM.docx]

**Supplementary Material**

**Supplemental Material 1: Format of the diagnostic reasoning (DxR) exam.**

Initial presentation information  Developing broad differential (list of 5) with expected physical exam finding and follow-up questions for each choice

Additional history  Refining differential (top 3) and providing justification

Physical exam  Proposing diagnostic tests

Diagnostic test results  Interpreting test results Proposing and justifying leading diagnosis

Pathophysiology, including correlation with foundational knowledge (e.g., physiological) Therapeutic decision-making and foundational knowledge (e.g. mechanism of action; select DxRs)

Legend: The DxR exam consists of five sequential components, with students advancing forward through each part; backward navigation is not permitted. As students answer questions and advance to the next part, they receive additional clinical information and new questions.

**Supplemental Material 2: Study Habit Survey**

| **Question** | **Answer Method** | **Answer Choices** |
| --- | --- | --- |
| Which of the following resources did you use when preparing for the DxR examination? | Select the top 4 | - Step 1 Review books - Step 2 Review books - Step 1 MCQ resources - Step 2 MCQ resources - Other MCQ resources - Lecture materials - Cases and/or readings used in the small group sessions in the course - Required readings for the lectures and/or labs in the course - Materials from Clinical Skills and/or other institution-offered courses - Other |
| Which of the following strategies did you use when preparing for the DxR examination? | Select all that apply | - Individual study - Group study - Reorganizing course content from the way it was presented - Self-created cases - Other |
| When organizing your study for the DxR examination, how frequently did you start with patient presentations (signs and symptoms), and then learn about the associated diseases? | 5-point Likert scale | 1 - Not at all  2 - A little bit  3 - Somewhat  4 - Quite a bit  5 - A tremendous amount |
| Which of the following resources did you use when preparing for the MCQ examination? | Select the top 4 | - Step 1 Review books - Step 2 Review books - Step 1 MCQ resources - Step 2 MCQ resources - Other MCQ resources - Lecture materials - Cases and/or readings used in the small group sessions in the course - Required readings for the lectures and/or labs in the course - Materials from Clinical Skills and/or other institution-offered courses - Other |
| Which of the following strategies did you use when preparing for the MCQ examination? | Select all that apply | - Individual study - Group study - Reorganizing course content from the way it was presented - Self-created cases - Other |
| When organizing your study for the MCQ examination, how frequently did you start with patient presentations (signs and symptoms), and then learn about the associated diseases? | 5-point Likert scale | 1 - Not at all  2 - A little bit  3 - Somewhat  4 - Quite a bit  5 - A tremendous amount |

**Supplementary Material 3: Exam Performance Comparison and Correlations**

| **End of Course Exam Pair** | **DxR Exam Topic** | **Exam Format** | | **P-value** | **Pearson Correlation Coefficient** |
| --- | --- | --- | --- | --- | --- |
|  |  | DXR  Mean (SD) | MCQ  Mean (SD) |  |  |
| 1 | Asthma 1 | 88.8 (6.4) | 77.0 (7.8) | 0.001 | 0.46 |
| 2 | Asthma 2 | 90.0 (2.1) | 81.0 (8.6) | 0.14 | 0.13 |
| 3 | COPD | 83.0 (2.6) | 78.0 (7.3) | <0.001 | 0.39 |
| 4 | NSTEMI | 77.9 (11.6) | 77.0 (7.8) | <0.001 | 0.38 |
| 5 | STEMI | 87.0 (3.2) | 78.0 (7.3) | <0.001 | 0.48 |
| 6 | UC | 95.2 (1.2) | 89.0 (5.3) | 0.14 | 0.13 |
| 7 | CD | 82.0 (2.3) | 88.0 (5.8) | 0.14 | 0.46 |
| 8 | Hepatitis | 79.0 (4.0) | 90.0 (5.9) | 0.003 | 0.26 |
| 9 | Swyer | 73.0 (5.0) | 87.0 (5.3) | <0.001 | 0.42 |
| 10 | PICA Stroke | 83.8 (8.8) | 83.8 (6.4) | <0.001 | 0.38 |
| 11 | AICA Stroke | 86.0 (7.4) | 85.0 (5.2) | <0.001 | 0.47 |
| 12 | MS | 84.0 (7.5) | 84.0 (5.2) | 0.003 | 0.34 |
| 13 | PCOS | 84.5 (5.6) | 86.0 (4.5) | 0.002 | 0.28 |
| 14 | PID 1 | 86.8 (6.2) | 88.0 (5.2) | 0.002 | 0.28 |
| 15 | PID 2 | 79.0 (3.2) | 88.0 (5.2) | 0.001 | 0.28 |
| All Exam Pairs | | 84.0 (7.5) | 84.0 (5.2) | 0.003 | 0.34 |

*Notes: Each exam pair consisted of a DxR and NBME-style MCQ examination administered to the same student cohort within the same course, either simultaneously or within 24 hours. Scores reflect percentage performance. P values represent results of paired t-tests comparing mean performance between DxR and MCQ examinations for each exam pair.* *Pearson correlation coefficients represent the strength of association between individual student performance on DxR and MCQ examinations within each exam pair. The “All Exam Pairs” row reflects pooled analysis across all assessments.*

*Abbreviations. COPD: chronic obstructive pulmonary disease; NSTEMI: Non-ST elevation myocardial Infarction; STEMI: ST-elevation myocardial infarction; UC: Ulcerative Colitis; CD: Crohn’s Disease; PICA: Posterior Inferior Cerebellar Artery stroke syndrome; AICA: Anterior Inferior Cerebellar Artery stroke syndrome; MS: Multiple Sclerosis; PCOS = Polycystic Ovarian Syndrome; PID: Pelvic Inflammatory Disease*
